# Soluble Guanylyl Cyclase Is Reduced in Airway Smooth Muscle Cells From a Murine Model of Allergic Asthma

**DOI:** 10.1097/WOX.0b013e318201d80b

**Published:** 2010-12-15

**Authors:** Fabiola Placeres-Uray, Ramona González de Alfonzo, Itala Lippo de Becemberg, Marcelo J Alfonzo

**Affiliations:** 1 Sección de Biomembranas, Instituto de Medicina Experimental, Cátedra de Patología General y Fisiopatología, Escuela Luís Razetti, Facultad de Medicina, Universidad Central de Venezuela, Apto 50587, Sabana Grande, Caracas, Venezuela

## Abstract

Airway remodeling plays an important role in the development of airway hyperresponsiveness in asthma. Muscarinic agonists such as carbamylcholine increased cyclic GMP (cGMP) levels in bovine tracheal smooth muscle strips, via stimulation of NO-sensitive soluble guanylylcyclase (NO-sGC), which is an enzyme highly expressed in the lungs. cGMP production, by activation of a NO-sGC, may contribute to airway smooth muscle relaxation. To determine whether the bronchoconstriction observed in asthma is accompanied by changes in this NO-sGC activity, we used a well-established murine model, ovalbumin-airway smooth muscle cells (OVA-ASMCs) of allergic asthma to evaluate such hypothesis. Histologic studies of trachea specimens showed the existence of inflammation, hyperplasia and tissue remodeling in OVA-ASMCs. Interestingly, cultured OVA-ASMCs showed lower GC basal activity than CONTROL-ASMCs. Also, we found that both OVA-ASMCs and CONTROL cells exposed to carbamylcholine and sodium nitroprusside and combinations of both drugs increased cGMP levels, which were inhibited by 1H-[1,2,4]oxadiazolo[4,3-] quinoxalin-1-one. All the experimental evidence suggests that NO-sGC activity is reduced in isolated ASMCss from experimental asthma murine model.

## 

Asthma is a chronic inflammatory disease that involves a reversible obstruction of the airways [1]. Asthma pathogenesis is characterized by the presence of the following factors: (1) chronic inflammation, (2) airway hyperresponsiveness, and (3) tissue remodeling. Furthermore, the remodeling process is associated with the development of bronchial hyper-reactivity. These elements together are responsible for reversible obstruction of the airways and the clinical expression of asthma [1-3].

This chronic inflammatory process leads to structural changes in the bronchial wall, involving hypertrophy and hyperplasia of smooth muscle cells of airways (ASMCs)[2-5] as described above. The role of ASMCs in airway remodeling is supported by increased muscle mass in the bronchial tree. In this regard, there have been numerous morphometric studies to test such an increase in muscle mass and to try to establish the pathophysiologic mechanism, either hyperplasia (increase in cell number) or hypertrophy (increase in cell volume) of ASMCs [6-12].

The cultured ASMCs under certain conditions may develop different phenotypes. The "contractile phenotype" has an intense staining for smooth muscle-specific contractile proteins showing few organelles. By contrast, "synthetic phenotype" exhibits increased mitogenic activity, and reduced staining for contractile proteins and more organelles biosynthesis [13]. Studies on phenotype modulation of smooth muscle cells led to the hypothesis that the phenomenon of "phenotypic plasticity" is not a simple artifact of cell culture but rather the ASMCs "in vivo" express a certain range of phenotypes. However, ASMCs either "in situ" or in culture should not be considered exclusively contractile or synthetic, but there is a balance between a heterogeneous population of synthetic and contractile cells [13].

NO is a bronchodilator and upregulation of its production in the absence of other inflammatory stimuli decreases airway resistance and responsiveness [14]. However, the role of NO in asthma is elusive, as it remains unclear whether the excessive NO production associated with this disease is protective or destructive for lung tissue. It is possible that excessive NO lung production induces a downregulation of a highly expressed soluble guanylyl cyclase (sGC) in lung, which may occur in asthma [15]. Previous reports have shown that either in the lungs of asthmatic patients or in animal models of asthma high levels of the enzyme inducible nitric oxide synthase (iNOS) has been expressed [16]. However, despite the presence of large amounts of NO that could activate sGC in smooth muscle and cause relaxation of the airways, it does not occur in asthmatic patients. One possibility is to determine whether the bronchoconstriction observed in asthma is related to sGC changing activity, which may exist in an allergic asthma model developed in murine (rats).

## Materials and methods

### Ovalbumin-Induced Rat Model of Asthma

Female Sprague-Dawley rats were sensitized with intraperitoneal injections of 10 *μg *of OVA (Sigma) plus 2 mg of aluminum hydroxide (Pharmacy School, UCV, Venezuela), as adjuvant, on days 0 and 5. From 7 to 14 days rats were continually exposed by nebulizer (DeVilbiss Pulmo-Aide model 5610D to an aerosol challenge containing either 0.9% saline or 1% OVA for 30 min/d as described previously [17].

### Isolation and Culture of Airway Smooth Muscle Cells

Primary cultures of rat ASMCs were established as previously reported [18]. Tracheas from rats were dissected in ice-cold phosphate-buffered saline (PBS) solution, pH 7.4. The epithelium was removed, and muscle layers were gently separated from underlying connective tissue in small bundles, which were placed in digestion solution containing 4 mg/mL collagenase II (Worthington) and Dispase (GIBCO) for 60 minutes at 37°C and 5% CO_2_. The cell suspension was centrifuged at 500 *g *for 15 minutes. Later, ASMCs were cultured in Dulbecco's modified Eagle's medium/F-12 (DMEM/F-12; GIBCO) supplemented with 10% fetal bovine serum (GIBCO), 1% L-glutamine, and 2% penicillin/streptomycin. These ASMCs were characterized[18] using immunofluorescence with the *α*-actin anti-smooth muscle monoclonal antibody (Sigma), being 100% positive after the third passage. ASMCs between passages 3 and 7 were used for experiments.

### Immunofluorescence Studies

The ASMCs in culture in cover slides were washed with saline and treated with a fixative solution (2% paraformaldehyde and 1% sucrose in PBS, pH 7.4) for 30 minutes at room temperature. Subsequently, the ASMCs were washed with PBS, permeabilized with increasing concentrations of methanol from 50% to 100% for 2 minutes at room temperature. Later, the cells were incubated for 1 hour in a buffer solution (PBS, 0.88% NaCl, and 2% bovine serum albumin) to saturate nonspecific binding sites. The ASMCs were incubated for 3 hours at 4°C with the primary antibody monoclonal anti-*α*-actin (Sigma) and specific smooth muscle in a dilution of 1:100 in PBS, 0.88% NaCl, and 2% albumin. Furthermore, a washing step with the same buffer and a second secondary antibody IgG (1:50 dilution) conjugated rabbit anti-mouse-1-fluorescein isothiocynate (FITC) labeling was performed by incubating for 30 minutes at room temperature. Finally, immunolabeled cells were washed with PBS for 20 minutes at room temperature. The fluorescence emitted from labeled cells was visualized in a fluorescence microscope (NiKon Labophot) at a magnification of × 100 and × 200 and photographic records were performed.

### cGMP Production of ASMCs

The ASMCs were incubated for 15 minutes in the presence of IBMX (100 *μ*M). cGMP production was determined in the presence of carbamylcholine (**C**ch, 1 × 10^-5 ^M), sodium nitroprusside (SNP, 100 *μ*M), and 1H-[1,2,4]oxadiazolo[4,3-]quinoxalin-1-one (ODQ, 100 nM) for 5 minutes at 37°C. The reaction was stopped by removing the medium immediately and freezing with liquid N_2_, and later 500 *μ*L of 6% trichloroacetic acid was mixed vigorously and the suspension was centrifuged for 1500 *g *× 15 minutes. The acid supernatants were extracted 2 times with ether saturated with water. Later, the aqueous solutions were lyophilized and resuspended in 150 *μ*L of water. The cGMP produced was determined by radioimmunoassay using the cGMP kit from Amersham, in 50 *μ*L of the supernatant, and the radioactivity [^3^H] was measured by liquid scintillation spectrometry. Total protein content of ASMCs was determined in the trichloroacetic acid pellet, which was dissolved in a small amount of 1 N NaOH and an aliquot was employed to estimate protein content, using Lowry procedure [19]. The amount of cGMP is expressed in pmol/mg of total cellular protein.

### Statistical Analysis

Data are mean ± SEM. The statistical significance of differences between means was determined by an unpaired two-tailed Student *t *test. Differences were considered to be significant (*P *< 0.05).

## Results and Discussion

### Histologic Evaluation of the Trachea of Sprague-Dawley Rats (CONTROL and Sensitized with OVA)

**Figure 1:**
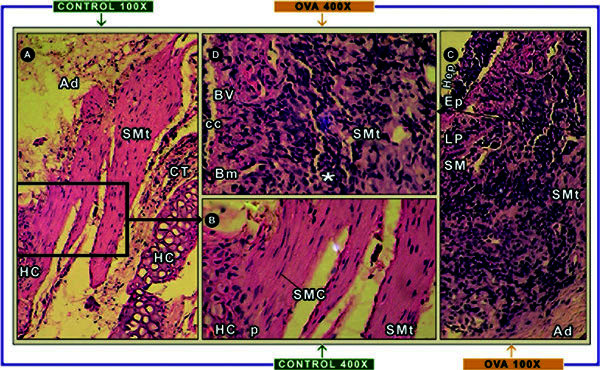
**Airway smooth muscle histologic studies from OVA-sensitized and control rats**. The panels (A) and (B) represent the typical tracheal tissue of the CONTROL rats group showing a thin layer of smooth muscle (SMt) located between the edges of the hyaline cartilage ring (HC) and it is enclosed by connective tissue (CT) with the adventitia. The SM is bound to perichondrial tissue (p) through bundles of smooth muscle cells (SMC). These SMC cells have elongated shape, eosinophilic cytoplasm, and basophilic nucleus. The panels (C) and (D) represent the tracheal wall of OVA rats, having a characteristic layered arrangement of the trachea, because the epithelium (Hep) is thickened with hyperplasia of caliciform cells (mL), basal membrane (Bm), lamina propia (LP), and tunic submucosa (SM). Increased cellular connective tissue and mononuclear cell infiltration (*), and formation of blood vessels (BV), are shown. In the layer of SM an inflammatory infiltrate around the SMC is also observed. Sections were stained with hematoxylin & eosin. Magnification: × 100 (A, C) and × 400 (B, D).

Histologic analyses confirmed the presence of inflammation, a hyperplasia and hypertrophy of smooth muscle tissue in the tracheas of OVA-sensitized rats as shown in Figure 1. An interstitial edema of the lamina propia and submucosa in the OVA group was observed. Also, an increase in cell amount of the connective tissue in close association with and hardly distinguishable from inflammatory infiltrate cells was observed. The hyperplastic tissue was located in the submucosa close to the perichondrial tissue. Also, there were elongated smooth muscle cells with a tendency to form bundles (Figures 1C, D). Inflammatory cell infiltrate being mainly mononuclear cells was present in mucosa, submucosa, and smooth muscle (Figures 1C, D). Angiogenesis was present in the lamina propria and submucosa, which were not observed in CONTROL rats' trachea (Figure 1). Thickness of the adventitia is due to greater deposition of extracellular matrix (Figure 1). Finally, the structure of the tunica tracheal cartilage of the OVA-rats group showed no difference with respect to CONTROL.

The histologic findings of the CONTROL rats' trachea compared with OVA rats indicate that OVA sensitization triggered an inflammatory process, which was associated with the remodeling of the tracheal wall, which is similar to ones that have been described in patients with human asthma [2-5]. In summary, in our murine (rat) model, tissue remodeling was present in all regions of the tracheal wall and was characterized by hyperplasia and denudation of the epithelial layer, subepithelial fibrosis, angiogenesis, and thickness of all layers. Interestingly, in OVA samples a mononuclear cell infiltrate was found in the mesenchymal tissue cells and smooth muscle cells.

### Immunofluorescence Studies

**Figure 2:**
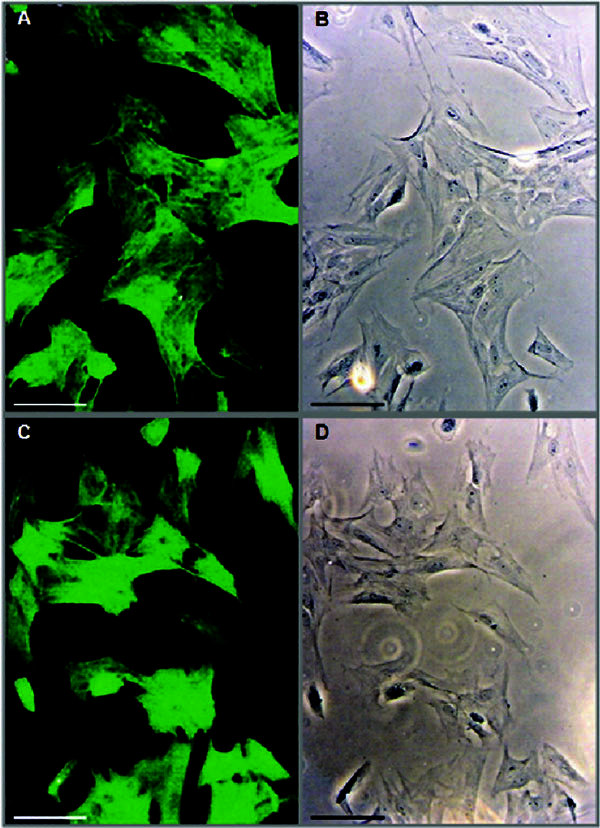
**Characterization of airway smooth muscle cells by *α*-actin smooth muscle immunofluorescence: Specific primary antibody against *α*-actin (1:100) and FITC-conjugated secondary antibody (1:50) were used**. Panels (C, OVA) and (A, CONTROL) represent the typical photography records of cells culture under fluorescence microscope. Panels (D, OVA) and (B, CONTROL) are phase-contrast microscopy records. Magnification: × 200. Bar = 150 *μ*m. The immunolabeling was performed in ASMCs from different trachae from OVA and CONTROL rats (*n *= 3) for each experimental condition.

The characterization of culture smooth muscle cells was performed by indirect immunofluorescence. The specific anti-*α*-actin antibodies immunolabeling was performed in primary cultures, revealing that 100% were positive for smooth muscle cells in both the OVA and CONTROL groups, though passages 3 and 5 (Figure 2) showed long, straight, uninterrupted fibrils densely arranged along its longitudinal axis. The nucleus and the cytoplasm located between the myofilaments were not labeled [20,21].

Consequently, the cells being positive with anti-*α*-smooth muscle actin indicates that isolated cells from all tracheas (OVA and CONTROL groups) correspond to ASMCs and not another cell type. In addition, our cultured ASMCs exhibited the classic modulation processes (transition to the synthetic-proliferative phenotype) and maturation (transition to the contractile phenotype) described in previous studies [13,22-24]. These results argue against the ASMC morphology heterogeneity described in primary cultures, which was explained by 2 different isolated cell types, or the presence of 2 phenotypes of smooth muscle cells, such as described elsewhere [20,21].

Interestingly, our primary cultures obtained from OVA group rats are composed of cells, exhibiting an ASMC synthetic-proliferative phenotype with an increment in density or confluence monolayer, giving the appearance of less organized culture compared with the CONTROL group.

### cGMP Production in Cultured ASMCs

Previous evidences suggest that excessive NO production induces a downregulation of NO-sensitive guanylyl cyclase (sGC), which may occur in asthma [15]. Following this rationale, we investigated the cGMP production in airway smooth muscle cultured cells in the presence of sGC-selective activators (SNP, a donor of NO) and a selective sGC inhibitor (ODQ). In addition, muscarinic agents such as Cch have been shown to increase the cGMP levels in intact tracheal smooth muscle strips, via sGC activation[25] and membrane-bound guanylylcyclases,[26] which was also evaluated in our cell culture assays.

**Figure 3:**
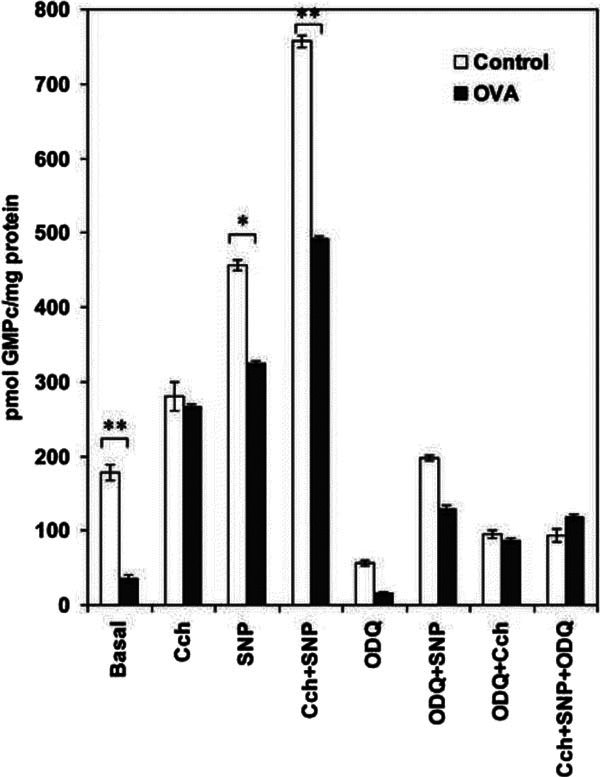
**cGMP production in ASMCs from CONTROL and OVA-sensitized rats**. The ASMCs were incubated for 15 minutes in the presence of IBMX as described in the Materials and Methods. cGMP production was determined in the presence of Cch, SNP, and ODQ as described in the Materials and Methods. The cGMP produced was estimated in duplicate using a radioimmunoassay kit from Amersham. Each value is the mean ± of 5 different experiments. In both groups, the number of cell culture plates was obtained for a pool of 5 rats. The statistical significance between CONTROL versus OVA was established as *P *< 0.05 (*) and *P *< 0.001 (**).

Thus, the sGC activity was determined, in cultured ASMCs, preincubated for 15 minutes with 100 *μ*M IBMX (a powerful inhibitor of cyclic nucleotide phosphodiesterase). Thus, the GC activity was estimated indirectly by the production of cGMP in the presence of 100 *μ*M SNP and 1 × 10^-5 ^M Cch and the combination of both agents. Thus, in ASMC CONTROL group (*n *= 5), the basal activity was stimulated >2.6 times by SNP, being increased 1.6 times with Cch and further potentiated synergistically (>4.2 times) in the combination of Cch and SNP. To establish the sGC role, a classic inhibitor of this NO-sensitive GC as ODQ was tested in all assay conditions. Thus, in CONTROL ASMCs, 100 nM ODQ inhibited the basal activity >70%, the SNP stimulation was decreased in 55%, and the Cch stimulation was decreased in 28%. All these data suggest that sGC was involved in such cGMP production. Interestingly, in OVA-ASMCs (*n *= 5) showed lower GC basal activity compared with CONTROL ASMCs (*P *< 0.05). In this sense, the fold stimulations induced by SNP (9 times) and Cch (7 times) and SNP + Cch (14 times) were higher in OVA-ASMCs than in CONTROL ASMCs, all being inhibited by ODQ in >70%. These results are shown in Figure 3.

Our results showed that NO-sensitive sGC activity is decreased in ASMCs from a well-established murine model of allergic asthma associated with rat sensitized and challenged with OVA. Our results in OVA-ASMCs support the experimental findings in intact lung tissue from OVA-sensitized mice described by Papapetropoulos et al.[15] These authors described in a experimental asthma murine model a substantial decrease between 60% and 80% in the steady-state levels of sGC subunit mRNA from lung tissue using real-time PCR. In addition, these changes in mRNA were paralleled by changes at the protein level expression, which was reduced by 50%-80% as determined by Western blotting [15].

Interestingly, our experimental results indicate that OVA-ASMCs retained the cell phenotype that exists in the intact murine lungs, demonstrating that our findings about the reduced sGC is not an artifact or an induced response of cultured ASMCs due to the tissue culture conditions. In addition, this experimental finding supports the use of this murine model for future studies in allergic asthma. At this moment, we do not have evidence to define the type of phenotype ("contractile" or "synthetic") displayed by our ASMCs responsible for these biologic responses. However, ASMCs either "in situ" or in culture should not be considered exclusively contractile or synthetic, but rather as a balance between a heterogeneous population of synthetic and contractile cells [13].

Interestingly, the NO-cGMP-dependent protein kinase cascade has been implicated in the regulation of the mitogen activated protein kinase (MAPK) system [27,28]. Thus, the decrement in this cyclic nucleotide cascade activity induces the activation of the MAPK kinases leading to cell division, which is associated with the hyperplasia of airway smooth muscle [28].

It is possible that a long-term downregulation of sGC in asthma may contribute to the increased airway reactivity to bronchoconstrictors and might also be implicated in the hyperplastic smooth muscle responses and remodeling that occurs in this lung disease.

## Conclusions

We found that all ASMCs exposed to a NO-donor compound as SNP and muscarinic agonist as carbamylcholine increased cGMP intracellular levels, which were inhibited by ODQ, suggesting that sGC is the main guanylyl cyclase enzyme responsible for cGMP production in these ASMCs. However, OVA-ASMCs showed low basal cGMP production compared with CONTROL ASMCs possibly because of reduced sGC expression. It is important to emphasize that the cGMP degradation was inhibited by the use of IBMX. In addition, SNP and Cch stimulation in OVA-ASMCs were higher than CONTROL ASMCs. Both these cGMP-dependent SNP and Cch increments in OVA-sensitive ASMCs were inhibited to ODQ.

We concluded that the ASMCs from OVA-sensitized rats display morphologic and proliferative characteristics different from the CONTROL as similar to ones described elsewhere in intact OVA-sensitized murine lungs [15]. Thus, both of the sGC activities expressed as the cGMP cell content were reduced in the experimental asthma model, which may contribute to airway hyperreactivity present in asthma.

## End note

Supported by grants from CDCH-UCV PG-09-00-7401-2008/1, CDCH-UCV PI-09-00-6464-2006/2 (R.A.G.), PI-09-7726-2009/1 (I.L.B.), and FONACIT S1-2002000411 (I.L.B.).

Presented at the World Allergy Congress 2009 meeting held December 6-10, 2009, Buenos Aires, Argentina.
